# X-Vectors: New Quantitative Biomarkers for Early Parkinson's Disease Detection From Speech

**DOI:** 10.3389/fninf.2021.578369

**Published:** 2021-02-19

**Authors:** Laetitia Jeancolas, Dijana Petrovska-Delacrétaz, Graziella Mangone, Badr-Eddine Benkelfat, Jean-Christophe Corvol, Marie Vidailhet, Stéphane Lehéricy, Habib Benali

**Affiliations:** ^1^Paris Brain Institute—ICM, Centre de NeuroImagerie de Recherche—CENIR, Paris, France; ^2^Laboratoire SAMOVAR, Télécom SudParis, Institut Polytechnique de Paris, Palaiseau, France; ^3^Sorbonne University, Inserm, CNRS, Paris Brain Institute—ICM, Paris, France; ^4^Assistance Publique Hôpitaux de Paris, Hôpital Pitié-Salpêtrière, Department of Neurology, Clinical Investigation Center for Neurosciences, Paris, France; ^5^Assistance Publique Hôpitaux de Paris, Hôpital Pitié-Salpêtrière, Department of Neuroradiology, Paris, France; ^6^Department of Electrical & Computer Engineering, PERFORM Center, Concordia University, Montreal, QC, Canada

**Keywords:** Parkinson's disease, x-vectors, voice analysis, early detection, automatic detection, telediagnosis, MFCC, deep neural networks

## Abstract

Many articles have used voice analysis to detect Parkinson's disease (PD), but few have focused on the early stages of the disease and the gender effect. In this article, we have adapted the latest speaker recognition system, called x-vectors, in order to detect PD at an early stage using voice analysis. X-vectors are embeddings extracted from Deep Neural Networks (DNNs), which provide robust speaker representations and improve speaker recognition when large amounts of training data are used. Our goal was to assess whether, in the context of early PD detection, this technique would outperform the more standard classifier MFCC-GMM (Mel-Frequency Cepstral Coefficients—Gaussian Mixture Model) and, if so, under which conditions. We recorded 221 French speakers (recently diagnosed PD subjects and healthy controls) with a high-quality microphone and via the telephone network. Men and women were analyzed separately in order to have more precise models and to assess a possible gender effect. Several experimental and methodological aspects were tested in order to analyze their impacts on classification performance. We assessed the impact of the audio segment durations, data augmentation, type of dataset used for the neural network training, kind of speech tasks, and back-end analyses. X-vectors technique provided better classification performances than MFCC-GMM for the text-independent tasks, and seemed to be particularly suited for the early detection of PD in women (7–15% improvement). This result was observed for both recording types (high-quality microphone and telephone).

## 1. Introduction

Parkinson's disease (PD) is the second most common neurodegenerative disease after Alzheimer's disease and affects approximately seven million people worldwide. Its prevalence in industrialized countries is around 0.3% and increases with age: 1% of people over the age of 60 and up to 4% of those over 80 are affected (De Lau and Breteler, 2006). The prevalence of PD has doubled between 1990 and 2016, which may be explained by the rise in life expectancy, better diagnoses and environmental factors. This disease results in motor disorders worsening over time caused by a progressive loss of dopaminergic neurons in the substantia nigra (located in the midbrain). The standard diagnosis is mainly based on clinical examination. Usually the diagnosis is made when at least two of the following three symptoms are noted: bradykinesia (slowness of movement), rigidity, and tremors at rest. Unfortunately, these motor symptoms appear once 50–60% of dopaminergic neurons in the substantia nigra (Haas et al., 2012) and 60–80% of their striatal endings (Fearnley and Lees, 1991) have degenerated. That is why detecting PD in the early stages remains a big challenge, in order to test treatments before the occurrence of large irreversible brain damage, and later to slow down, or even stop, its progression from the beginning.

Voice impairment is one of the first symptoms to appear. Many articles have used voice analysis to detect PD. They observed vocal disruptions, called hypokinetic dysarthria, expressed by a reduction in prosody, irregularities in phonation, and difficulties in articulation. The classification performances (accuracy rate) using voice analysis ranged from 65 to 99% for moderate to advanced stages of the disease (Guo et al., 2010; Rustempasic and Can, 2013; Shahbakhi et al., 2014; Ozkan, 2016; Gómez-Vilda et al., 2017; Ali et al., 2019; Avuçu, 2020). Fewer studies focused on early detection of PD through voice. Moreover, they usually worked on rather small databases (around 40 subjects) and analyzed men or mixed-gender groups (Rusz et al., 2011, 2015b; Novotný et al., 2014; Orozco-Arroyave et al., 2016a). Recently, PD detections using telephone networks have been carried out, from controls and early PD subjects selected by neurologists (Jeancolas et al., 2019), as well as from self-selected participants at any stage of the disease (Arora et al., 2019).

Different classification methodologies have been explored to detect PD using voice analysis. The first studies used global features, such as the number of pauses, the number of dysfluent words, the standard deviation (SD) of pitch and of intensity, along with averaged low-level perturbations, such as shimmer, jitter, voice onset time, signal to noise ratio, formants, or vowel space area, which are reviewed in Jeancolas et al. (2016). The authors usually performed a feature selection, keeping statistically significant features and removing the redundancies. Finally, selected features were fed to classifiers, such as Support Vector Machines (SVM) (Gil and Johnson, 2009; Little et al., 2009; Rusz et al., 2011, 2015a; Sakar et al., 2013, 2017; Novotný et al., 2014), k-nearest neighbors (Sakar et al., 2013, 2017), decision trees (Mucha et al., 2017), multilayer perceptrons (Gil and Johnson, 2009), probabilistic neural networks (Ene, 2008), or minimax classifiers with gaussian kernel density (Rusz et al., 2013).

Another type of features, which has been used in the field of speaker recognition for decades, is the Mel-Frequency Cepstral Coefficients (MFCCs) (Bimbot et al., 2004) (for abbreviations and definitions see Table 1). These short-term features, calculated on (20–40 ms) windows, characterize the spectral envelope, and reflect the shape of the vocal tract (composed of three connected cavities: pharyngeal, oral, and nasal). Several muscles and articulators, such as the pharyngeal constrictor muscles, tongue, lips, jaw, larynx, soft palate, and larynx location, modulate the shapes, volumes, and the coupling of these cavities, giving a specific timbre to the sound. This results in a particular spectral envelope, described by the MFCCs. Thus, MFCCs extracted throughout the vocal tasks, capture information related to articulation and phonation.

**Table 1:** List of abbreviations.

**Abbreviation**	**Definition**
PD	Parkinson's disease
HC	Healthy control
SD	Standard deviation
MFCC	Mel-frequency cepstral coefficients
GMM	Gaussian mixture model
MDS-UPDRS	Movement disorder society sponsored revision of the unified Parkinson's disease rating scale
LLH	Log-likelihood
DNN	Deep neural network
TDNN	Time delay neural network
LDA	Linear discriminant analysis
PLDA	Probalistic linear discriminant analysis
DDK	Diadochokinesia
EER	Equal error rate
DET	Detection error tradeoff

Over the past 15 years, MFCCs have appeared in the detection of vocal pathologies, such as dysphonia (Dibazar et al., 2002; Godino-Llorente and Gómez-Vilda, 2004; Malyska et al., 2005). The use of MFCCs for PD detection was introduced in Tsanas et al. (2012). Since then, many studies have used MFCCs for PD detection (Arias-Vergara et al., 2017; Naranjo et al., 2017; Vaiciukynas et al., 2017; Drissi et al., 2019; Fang et al., 2020) or PD monitoring (Grosz et al., 2015; Schuller et al., 2015; Orozco-Arroyave et al., 2016b).

Several statistical analyses and classifiers can be applied on MFCC features. For instance, if MFCC dispersion is low within classes, generally due to a poor phonetic variety, one can simply consider the MFCC averages (in addition to other features). This is generally the case for sustained vowel tasks (Tsanas et al., 2012; Jafari, 2013; Benba et al., 2014, 2016; Orozco-Arroyave et al., 2015a; Hemmerling et al., 2016) or when phonetically similar frames are selected (Orozco-Arroyave et al., 2014, 2015b, 2016a). Authors often add to the means some other statistics like the standard deviation, kurtosis (flattening measurement), and skewness (asymmetry measurement) in order to gain a little more information. These features are then fed into classifiers such as SVMs, multilayer perceptrons, or decision trees.

If frames are acoustically very different (such as during reading or free speech tasks), additional precision is required to describe the MFCC distribution. One possible modeling technique uses vector quantization (Kapoor and Sharma, 2011; Benba et al., 2014). Another more precise way is to model the MFCC distribution with a Gaussian Mixture Model (GMM). GMM can model the MFCC distribution of the PD and control groups. Likelihood scores of test subjects' MFCCs against the two GMM models (PD and control) are then calculated (Moro-Velázquez et al., 2018; Jeancolas et al., 2019). GMM can also model the MFCC distribution of each subject. The means of the Gaussian functions (forming a “supervector”) are then fed into a classifier such as SVM (Bocklet et al., 2013). When not enough speech data is available to train the GMM models, which mainly occurs when GMMs are used to model each subject (rather than a group), GMMs can be adapted from Universal Background Models (UBM) previously trained with a bigger dataset (Reynolds et al., 2000; Bocklet et al., 2013). More than that, a more recent speaker recognition technique, called i-vectors, has been adapted for PD detection (Garcia et al., 2017; Moro-Velázquez et al., 2018). This approach consists in removing the UBM mean supervector and projecting each supervector onto a lower dimensional space, called the total variability space. Intra-class variability is then often handled by means of discriminant techniques, like Linear Discriminant Analysis (LDA), or Probabilistic Linear Discriminant Analysis (PLDA). In PD detection this results in compensating the speaker, channel, and session effects. In López et al. (2019), the authors compared the i-vectors system with another MFCC-based speaker representation, using Fisher vectors, and found superior PD detection performance for the latter.

Over the last few years, with the increase of computing power, several Deep Neural Network (DNN) techniques have emerged in PD detection. Some studies applied Convolutional Neural Networks on spectrograms (Vásquez-Correa et al., 2017; Khojasteh et al., 2018; Zhang et al., 2018). Others used DNNs to extract phonological features from MFCCs (Garcia-Ospina et al., 2018), or to detect directly PD from global features (Rizvi et al., 2020).

In the present study, we adapted a brand-new text-independent (i.e., no constraint on what the speaker says) speaker recognition methodology, introduced in Snyder et al. (2016). This approach consists in extracting embedding features (called x-vectors) from a DNN taking MFCCs as inputs.

According to the authors, the advantages of x-vectors are that they capture well the characteristics of speakers that have not been seen during the DNN training, that they provide a more robust speaker representation than i-vectors (Snyder et al., 2017), and that they improve speaker recognition, provided that a large amount of training data is available (Snyder et al., 2018b).

In 2018, the same authors adapted the x-vector method to language recognition (Snyder et al., 2018a) and outperformed several state-of-the-art i-vector systems.

Recently, we proposed an adaptation of x-vectors for PD detection in Jeancolas (2019). Since then, another work has used x-vectors for PD detection (Moro-Velazquez et al., 2020). In this paper we made different experimental choices. Unlike (Moro-Velazquez et al., 2020), we focused on PD detection at an early stage, and performed the classifications on high-quality recordings on the one hand and on telephone recordings on the other hand. We also tested different types of speech tasks (text-dependent and text-independent) and different datasets for the DNN training, in order to assess their impact on PD detection. In order to achieve the best performance, we also considered men and women separately. This is usually done in speaker recognition and has been proved to enhance vocal pathology detections involving MFCC features (Fraile et al., 2009). Moreover, this allowed us to analyze the effect of gender on PD detection. We also made different methodological choices. We studied the effect of important x-vectors methodological aspects, such as the audio segment durations and data augmentation. Finally we assessed the advantage of considering an ensemble method for the classification. For each condition, we compared different classifiers: cosine similarity (with and without LDA) and PLDA, which are commonly used with x-vectors, and as a baseline, the MFCC-GMM technique we used in Jeancolas et al. (2019).

## 2. Materials and Methods

### 2.1. Databases

#### 2.1.1. Participants

A total of 221 French speakers were included in this study: 121 PD patients and 100 healthy controls (HC). All PD patients and 49 HC were recruited at the Pitié-Salpêtrière Hospital and included in the ICEBERG cohort, a longitudinal observational study conducted at the Clinical Investigation Center for Neurosciences at the Paris Brain Institute (ICM). An additional 51 HC were recruited to balance the number of PD and control subjects. All patients had a diagnosis of PD, according to the United Kingdom Parkinson's Disease Society Brain Bank (UKPDSBB) criteria, <4 years prior to the study. HC were free of any neurological diseases or symptoms. Participants had a neurological examination, motor and cognitive tests, biological sampling, and brain MRI. PD patients were pharmacologically treated and their voices were recorded during ON-state (<12 h after their last medication intake). Data from participants with technical recording issues, language disorders not related to PD (such as stuttering) or when a deviation from the standardized procedure occurred, were excluded from the analysis. The ICEBERG cohort (clinicaltrials.gov, NCT02305147) was conducted according to Good Clinical Practice guidelines. All participants received informed consent prior to any investigation. The study was sponsored by Inserm, and received approval from an ethical committee (IRBParis VI, RCB: 2014-A00725-42) according to local regulations.

#### 2.1.2. High-Quality Microphone Recordings

Among the 217 participants kept for the analysis, 206 subjects including 115 PD (74 males, 41 females) and 91 HC (48 males, 42 females) performed speech tasks recorded with a high-quality microphone. Information about age, time since diagnosis, Hoehn and Yahr stage (Hoehn and Yahr, 1967), Movement Disorder Society-sponsored revision of the Unified Parkinson's Disease Rating Scale (MDS-UPDRS) III score (Goetz et al., 2007) (OFF state) and Levodopa Equivalent Daily Dose (LEDD) are detailed in Table 2. The microphone was a professional head mounted omnidirectional condenser microphone (Beyerdynamics Opus 55 mk ii) placed approximately 10 cm from the mouth. This microphone was connected to a professional sound card (Scarlett 2i2, Focusrite) which provided phantom power and pre-amplification. Speech was sampled at 96,000 Hz with 24 bits resolution and a frequency range of 50 Hz–20 kHz. ICEBERG participants were recorded in consultation rooms in the Clinical Investigation Center of the Paris Brain Institute or in the Sleep Disorders Unit of the Pitié-Salpêtrière hospital in Paris. Additional HC were recorded in quiet rooms in their own house or at their office with the same recording devices. Speech tasks were presented in a random order to the participants via a graphical user interface. The tasks which are analyzed in the present study are: reading (1 min), sentence repetition (10 s), free speech (participants were asked to talk about their day during 1 min) and fast syllable repetitions (1.5 min), also called diadochokinesia (DDK) tasks. Details about speech task content are presented in Annex 1.

**Table 2:** High-quality microphone database information.

	**Number**	**Age (years)**	**Disease duration (years)**	**H & Y**	**MDS-UPDRS III**	**LEDD (mg)**
		**Mean ± SD**	**Mean ± SD**	**Mean ± SD**	**Mean ± SD**	**mean ± SD**
**PD**	**115**	**63.8 ± 9.3**	**2.6 ± 1.5**	**2.0 ± 0.1**	**32.5 ± 7.0**	**392 ± 266**
M	74	63.7 ± 9.3	2.5 ± 1.4	2.0 ± 0.1	34.1 ± 7.0	415 ± 298
F	41	63.9 ± 9.3	2.7 ± 1.5	2.0 ± 0.0	29.6 ± 5.8	352 ± 191
**HC**	**91**	**59.1 ± 10.0**	–	**0.0 ± 0.3**	**4.8 ± 3.5**	–
M	48	58.9 ± 10.7	–	0.0 ± 0.0	4.6 ± 3.7	–
F	43	59.3 ± 9.2	–	0.1 ± 0.4	4.9 ± 3.4	–
**Total**	**206**	**61.7 ± 9.8**	–	**1.5 ± 0.9**	**24.8 ± 13.9**	–

#### 2.1.3. Telephone Recordings

Most of the participants, 101 PD (63 males, 38 females) and 61 HC (36 males, 25 females) also carried out telephone recordings. Information about age, time since diagnosis, Hoehn and Yahr stage, MDS-UPDRS III score (OFF state), and LEDD are detailed in Table 3. Participants called once a month an interactive voicemail (IVM, from NCH company), connected to a SIP (Session Initiation Protocol) server (ippi), with their own phone (mobile or landline). Audio signal was compressed with G711 codec and transformed into PCM16 audio files by IVM. Finally, speech files were sampled at 8,000 Hz with 16 bits resolution, and a frequency bandwidth of 300–3,400 Hz. We set up the voicemail to automatically make the participants carry out a set of speech tasks when they called. Participants performed different numbers of recording sessions (from 1 to 13 with an average of 5) depending on when they started and stopped. The tasks that we analyzed in this study were: sentence repetition (20 s), free speech (1 min) and DDK tasks (1 min). For practical reasons, only audio instructions were given during the phone calls. Therefore no reading tasks were performed. Details about speech task content are presented in Annex 1, and transmission chain and encoding are described in Annex 2.

**Table 3:** Telephone database information.

	**Number**	**Age (years)**	**Disease duration (years)**	**H & Y**	**MDS-UPDRS III**	**LEDD (mg)**
		**Mean ± SD**	**Mean ± SD**	**Mean ± SD**	**Mean ± SD**	**Mean ± SD**
**PD**	**101**	**63.5 ± 9.0**	**2.6 ± 1.4**	**2.0 ± 0.1**	**32.4 ± 7.0**	**387 ± 272**
M	63	63.7 ± 9.0	2.5 ± 1.4	2.0 ± 0.1	34.2 ± 6.9	403 ± 311
F	38	63.3 ± 9.3	2.7 ± 1.5	2.0 ± 0.0	29.5 ± 6.1	359 ± 194
**HC**	**61**	**62.6 ± 8.5**	–	**0.0 ± 0.3**	**4.9 ± 3.5**	–
M	36	63.1 ± 9.3	–	0.0 ± 0.0	4.6 ± 3.5	–
F	25	61.8 ± 7.4	–	0.1 ± 0.5	5.3 ± 3.6	–
**Total**	**162**	**63.2 ± 8.9**	–	**1.4 ± 0.9**	**23.9 ± 14.1**	–

### 2.2. Methods

#### 2.2.1. Baseline: MFCC-GMM Methodology

In this section we present our MFCC-GMM baseline framework. This method, based on Gaussian mixture models fitting cepstral coefficients distributions of each class, has been used for decades in speaker recognition and was recently adapted for early PD detection (Jeancolas et al., 2019).

##### 2.2.1.1. Preprocessing and MFCC Extraction

The first preprocessing regarding our high-quality microphone recordings was spectral subtraction (Boll, 1979). The aim of this denoising technique was to compensate for the mismatched recording locations by removing additive and stationary noises. We applied it with the Praat software (Boersma, 2001), using the 5 s long silence recorded at the end of each participant's session for the calibration. Regarding the telephone recordings, spectral subtraction was not performed because acoustic environments of PD subjects and HC were alike.

We then extracted the log-energy and 19 MFCCs, using the Kaldi software (Povey et al., 2011), on 20 ms overlapping windows, with a 10 ms step. For the high-quality recordings, the 23 Mel-spaced triangular filters covered a frequency range of 20–7,000 Hz. As for the telephone recordings, the frequency range of the filters was 300–3,700 Hz. More details about the MFCC extraction methodology can be found in Jeancolas (2019). The first derivatives (Deltas) and second derivatives (Delta-Deltas) were then computed and added to the feature vectors, in order to provide additional speech dynamic information.

Once the MFCCs and their deltas were extracted, we carried out Vocal Activity Detection (VAD), based on the log-energy, in order to remove silent frames.

Finally, to complete denoising, a cepstral mean subtraction (Quatieri, 2001) was performed on 300 ms sliding windows, reducing linear convolutional channel effects on both databases.

##### 2.2.1.2. Distribution Modeling With Gaussian Mixture Models

We split the databases into three groups per gender: one group of PD subjects and one group of controls for training, and a third group for testing, containing all the remaining PD and control participants. In the laboratory setting database, we took 36 PD and 36 HC for the male training groups and 38 PD and 12 HC for the male test group. As for women, we considered 30 PD and 30 HC for training and 11 PD and 13 HC for testing. For the telephone database, we selected 30 PD and 30 HC for the male training groups and 33 PD and 6 HC for the male test group. For females we used 20 PD and 20 HC for training and 18 PD and 5 HC for the test. In order to have accurate and generalizable classification performances, the splits were repeated 40 times with the ensemble method described below.

**Figure 1:**
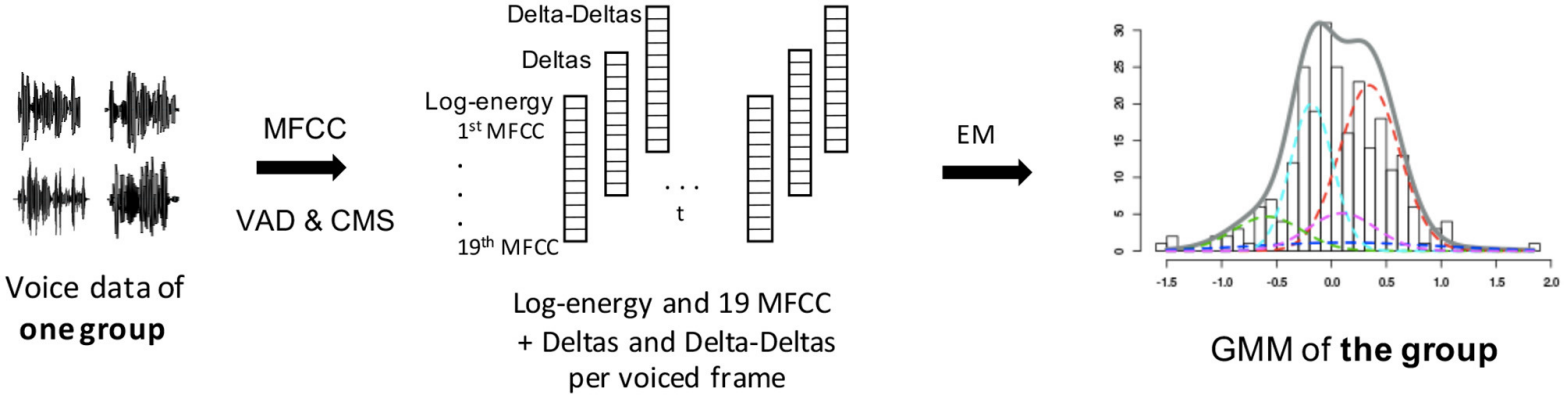
MFCC-GMM training phase: GMM training from MFCCs of each training group. The different colors of the GMM represent the different gaussian functions that compose it. The final GMM (gray curve) models the MFCC distribution of one training group (either male PD, female PD, male control, or female control). VAD, voice activity detection; CMS, cepstral mean subtraction; EM, expectation-maximization.

During the training phase, we built multidimensional GMMs, with the Kadi software, to model the MFCC distributions of each training group (see Figure 1). The means, SD, and weights of the Gaussians (characterizing the GMMs) were estimated via an Expectation-Maximization algorithm. The optimal number of Gaussian functions depends on the quantity of speech data used for training. We chose 20 Gaussian functions for the present analyses on the high-quality microphone database and 50 for the telephone database, as more sessions per subject were available.

##### 2.2.1.3. Classification

**Figure 2:**
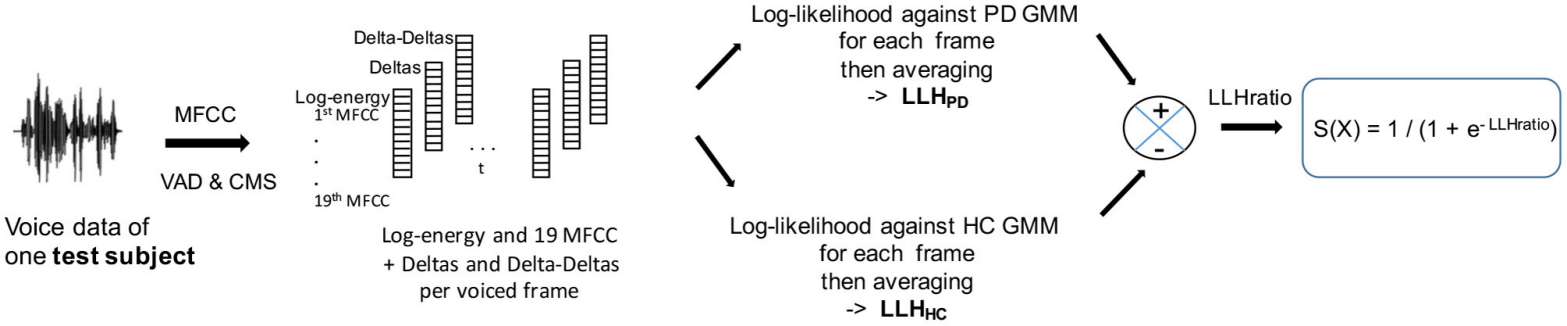
MFCC-GMM test phase: the test subjects' MFCCs are tested against a PD GMM model and a HC GMM model. The sigmoid of the log-likelihood ratio provides the classification score. VAD, voice activity detection; CMS, cepstral mean subtraction; LLH, log-likelihood.

For each test subject we calculated the log likelihood (LLH) of their MFCCs compared to the two GMM models corresponding to their gender. We first computed one log-likelihood per frame (after silence removal) of the test subjects' MFCCs against the two models, then we took the average over all the frames. Thus, the likelihood was guaranteed to be independent of the number of frames. A sigmoid function was then applied to the difference of these means (the *log-likelihood ratio*), so as to produce a score ranging from 0 to 1 per test subject (see Figure 2). A score closer to 1 indicated that the participants being tested could likely be associated to the PD condition, and a score closer to 0 that they could likely be associated to the HC condition.

##### 2.2.1.4. Validation and Ensemble Method

**Figure 3:**
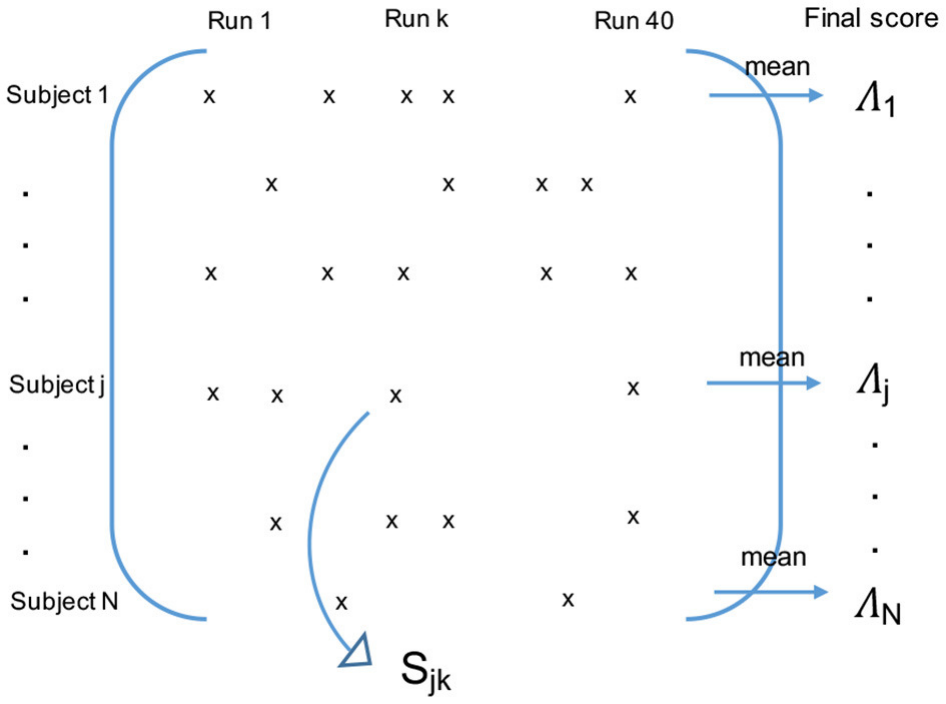
Final classification using the repeated random subsampling aggregation ensemble method. If the subject *j* belonged to the test group during the run *k*, *S*_*jk*_ is his/her intermediate classification score for this run. The final classification score Λ_*j*_ of the subject *j* is the average of his/her intermediate scores *S*_*jk*_ over the runs when he/she belonged to the test group.

Ensemble methods are techniques that create multiple models (in our case 40 × 2 GMMs) and then combine them to improve classifications or regressions. Ensemble methods usually produce more accurate solutions than a single model would (in our case one PD GMM and one HC GMM). That is why we chose to carry the final classification with an ensemble method. More precisely, we performed a repeated random subsampling aggregation (Bühlmann and Yu, 2002; Maillard et al., 2017), which is a type of bootstrap aggregation (Breiman, 1996) without replacement. We ran 40 times the GMM modeling and classification phases, each time with a different random split of participants between the training and test groups. The numbers of subjects per group were the ones previously stated. At the end of the 40 runs, all the subjects were tested about ten times. For each subject, we finally averaged the classification scores obtained during the runs when they belonged to the test group (see Figure 3).

The choice of this ensemble method was based on several elements:

- First of all, regarding the sampling technique, we chose repeated random subsampling rather than k-fold or Leave-one-subject-out (which are more common) because it allowed us to have the same number of PD and HC subjects for training. This led to same training conditions for the PD and HC GMMs, like same optimal number of Gaussians, therefore fewer hyperparameters and a reduced risk of overfitting.
- We then chose to carry the final classification with an ensemble method because they are known to decrease the prediction variance, usually leading to a better classification performance (Friedman et al., 2001).
- Regarding the type of aggregation, we chose to average the scores rather than use a majority vote type because it is the technique which is known to minimize the variance the most (Friedman et al., 2001).
- The error calculated on the final scores (of *out-of-bag* type) is known to be a good unbiased estimate of the real (or generalized) error, namely the one we would have if we tested an infinity of other new subjects on our aggregated model.

In section 3.7, we compared the classification performance of the aggregated model with the performance of the single model. The real (or generalized) performance of the single model (the one we would have if we tested an infinity of other new subjects against one PD GMM and one HC GMM trained with our current database) was estimated by the performance of the repeated random subsampling cross-validation (i.e., the average of the classification performance of each run). In all other sections we used the aggregated model for the classification.

#### 2.2.2. X-Vector Methodology

In this section we present the x-vector system we adapted from the latest speaker recognition method (Snyder et al., 2018b). X-vectors are fixed-length representations of variable-length speech segments. They are embeddings extracted from a DNN taking MFCC vectors as input, and are known to capture well speaker characteristics, even when the speakers have not been seen during the DNN training. Once the x-vectors had been extracted, we classified them according to the PD status of the related speaker, with different classification methods (cosine similarity, LDA + cosine similarity, and PLDA).

##### 2.2.2.1. DNN Training

Since DNN training usually requires a lot of data, we used a DNN trained on large speaker recognition databases and available online (http://kaldi-asr.org/models.html).

For the analysis of our telephone recordings, we considered the pretrained DNN SRE16 model, described in Snyder et al. (2018b). This DNN was trained on 5,139 subjects from the Linguistic Data Consortium catalog databases, including the Switchboard (Phase1,2,3 and Cellular 1,2), Mixer 6 and NIST SRE corpora. These databases contain telephone conversations and data recorded with a microphone, with English as the dominant language. Some data were directly sampled at 8 kHz, and the 16 kHz sampled recordings were then downsampled to 8 kHz.

For the analysis of our high-quality microphone recordings, we used the voxceleb model, trained on the voxceleb database (Nagrani et al., 2017). Data came from video interviews of 7330 celebrities posted on Youtube. Audio data was sampled at 16 kHz.

Finally, data augmentation, as described in section 2.2.2.3, was applied to all these DNN training datasets.

**Figure 4:**
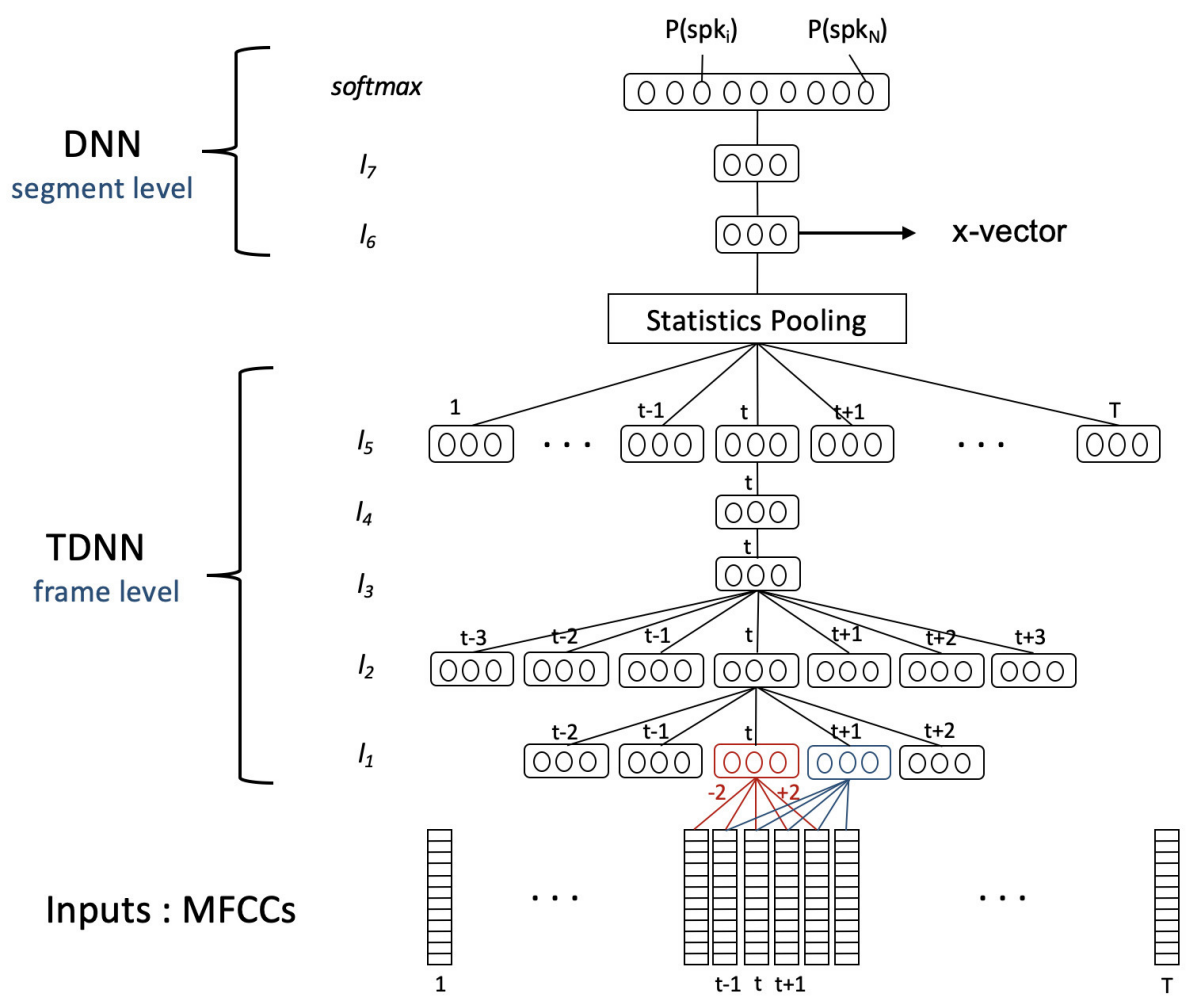
DNN diagram: training phase. The DNN was trained in the context of speaker identification. The goal was to identify speakers among the *N* training subjects (5,139 subjects for SRE16 and 7,330 for voxceleb) using their MFCCs extracted from 25 ms frames of a [2–4 s] audio segment (containing T frames). The statistics pooling layer aggregates the output of the TDNN network across the audio segment. The outputs of the last layer (softmax layer) are the probabilities that the audio segment belonged to each training speaker. X-vectors are the embeddings extracted from the first segment-level layer of the DNN. They are a representation of the audio segment and are a representation of a speaker when they are averaged over different audio segments of the same speaker.

These DNNs were trained in the context of speaker identification (see Figure 4), meaning the weights corresponding to the DNN different neurons were estimated so as to discriminate and identify the speakers among the training subjects (5,139 subjects for SRE16 and 7,330 for voxceleb) from their audio inputs. Inputs were the log energy and MFCCs extracted every 10 ms, from 25 ms windows of 2–4 s audio segments. For the SRE16 model, 23 MFCCs were extracted with a Mel filterbank range of 20–3,700 Hz. For the voxceleb model, 30 MFCCs were extracted with a filterbank range of 20–7,600 Hz. As for the MFCC-GMM analysis, a voice activity detection and cepstral mean subtraction were performed. This time, Deltas and Delta-Deltas were not computed because the temporal context was already taken into account in the temporal delay part of the DNN.

The architecture of the DNN is detailed in Table 4. The neural networks were composed of three parts:

– A set of frame-level layers taking MFCCs as inputs. These layers composed a Time Delay Neural Network (TDNN) taking into account a time context coming from neighboring frames.
– A statistics pooling layer aggregating the outputs (taking the mean and SD) of the TDNN network across the audio segment. The output of this step was a large-scale (3,000 dimensions) representation of the segment.
– The last part was a simple feed forward network composed of two segment-level layers taking as input the result of the pooling layer, reducing its dimensionality to 512 (providing the so-called x-vectors), and ending with a softmax layer. The softmax layer yielded the probability of the input segment coming from each speaker in the training database.

**Table 4:** DNN architecture.

**Layer**	**Frames**	**Input dim**	**Output dim**
Frame-level 1	5	5 × K	512
Frame-level 2	9	1,536	512
Frame-level 3	15	1,536	512
Frame-level 4	15	512	512
Frame-level 5	15	512	1,500
Pooling	T	1,500 × T	3,000
Segment-level 6	T	3,000	512
Segment-level 7	T	512	512
softmax	T	512	N

*X-vectors are extracted at layer segment-level 6 before the Rectified Linear Unit (ReLU) activation function. T is the number of frames composing the input segment. K corresponds to the number of input features for one frame, K = 24 for the telephone recordings (23 MFCCs + log energy) and K = 31 for the high-quality recordings (30 MFCCs + log energy). N is the number of speakers used for training, N = 5,139 for the SRE16 DNN and N = 7,330 for the voxceleb DNN*.

Language mismatch between DNN training and x-vector extraction is not an issue: x-vectors have been reported to be robust to this domain mismatch in speaker recognition (Snyder et al., 2017).

For the results presented in section 3.6, we trained a DNN with our own data (telephone recordings). The only difference in the DNN architecture was the size of the softmax layer output, which was two. Indeed, here the DNN was trained directly to discriminate PD subjects from HC (two classes) instead of discriminating between speakers (N classes).

##### 2.2.2.2. X-Vector Extraction

In order to extract the x-vectors for each subject of our databases we had to extract the MFCCs in the same way as it was done for the pretrained DNN. We extracted the log energy and 23 MFCCs every 10 ms for our telephone recordings (like the SRE16 model) and 30 MFCCs with log energy for our high-quality recordings (like the voxceleb model). For the high-quality microphone recordings, we first had to downsample them to 16 kHz (from 96 kHz), in order to match the sampling frequency used for the DNN training. Moreover, for this database as for the MFCC-GMM analysis, we carried out spectral subtraction to compensate for mismatched background noises. Voice activity detection and cepstral mean subtraction were also performed on both databases, as done for the SRE16 and voxceleb models and for our MFCC-GMM analysis.

X-vectors were then extracted for each subject. They were defined as the 512-dimensional vector extracted after the first segment-level layer of the DNN, just before the Rectified Linear Unit (ReLU) activation function.

Even if the audio segment tested did not belong to any speaker used to train the DNN, the x-vectors extracted could be considered as a representation of this segment and captured the speaker characteristics (Snyder et al., 2018a). Back-end analyses could then be carried out to classify the x-vectors corresponding to our participants, according to their PD status.

The audio segments used for the DNN training had a duration of 2–4 s (after silence removal). The DNN could be used to extract x-vectors from new unseen audio segments with durations comprised between 25 ms and 100 s. The audio segments of our database shorter than 25 ms were removed and the one longer than 100 s were divided into fragments smaller than 100 s. X-vectors corresponding to these fragments were then averaged.

We assessed the impact of matched segment durations between training and test in section 3.1. For all the other experiments we chose to divide our audio files into 1–5 s segments.

##### 2.2.2.3. Data Augmentation

In recent studies, speaker recognition using i-vectors and x-vectors has been enhanced by augmenting the data (Snyder et al., 2018b) for the DNN and PLDA trainings. Data augmentation consisted in duplicating the data, superposing an additive noise and reverberation effects on data copies. This led to increased quantity and diversity of samples available for the training. In our analyses, data augmentation was performed during the DNN training and we assessed its effect on the LDA and PLDA trainings. We used four different types of data augmentation:

– Reverberation: a reverberation was simulated by taking the convolution of our data with a Room Impulse Response (RIR) of different shapes and sizes, available online (http://www.openslr.org/28).
– Additive noise: different types of noise, extracted from the MUSAN database (http://www.openslr.org/17), were added every one second.
– Additive music: musical extracts (from the MUSAN database) were added as background noise.
– Babble: three to seven speakers (from the MUSAN database) were randomly selected, summed together, then added to our data.

The MUSAN and RIR NOISES databases were sampled at 16 kHz, so we downsampled them to 8 kHz for the telephone recordings analysis.

At the end, two out of the four augmented copies were randomly picked and added to our training database, multiplying by three its size.

##### 2.2.2.4. Back-End Analyses

**Figure 5:**
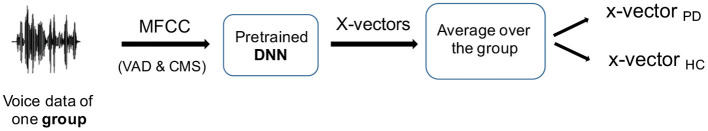
Reference x-vectors: x-vectors are computed for all the training subjects using their MFCCs, then averaged within the training groups (male PD, female PD, male control, and female control) in order to have one average x-vector per group. VAD, voice activity detection; CMS, cepstral mean subtraction; DNN, deep neural network.

Once the x-vectors were extracted for each subject, the x-vectors of the PD training group and the x-vectors of the HC training group were averaged in order to have one average x-vector representing each class, for each gender (see Figure 5).

**Figure 6:**
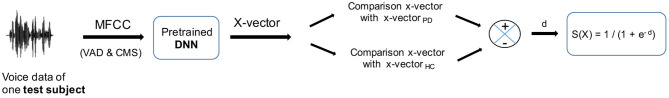
x-vector test phase: x-vectors are computed for each test subject from their MFCCs, then compared to the average x-vector_*PD*_ and x-vector_*HC*_. For the comparison we used cosine similarity (alone or after LDA projection) and PLDA. The sigmoid of the difference between similarity scores provides the classification score. VAD, voice activity detection; CMS, cepstral mean subtraction; DNN, deep neural network.

Classification of test subjects was done by comparing their x-vectors to the average x-vector_*PD*_ and x-vector_*HD*_, using a similarity measure. The difference between these two similarity measures was then calculated and normalized with a sigmoid function, providing a classification score between 0 and 1 per x-vector (see Figure 6). When there were several audio segments for a test subject, i.e., several x-vectors, the average of the classification scores of all the x-vectors was calculated. All the participants were split into training and test groups the same way as for the MFCC-GMM analysis.

Several methods exist to measure similarity between vectors. We compared three methods often used with i-vectors and x-vectors: cosine similarity, cosine similarity preceded by LDA, and PLDA.

*2.2.2.4.1. Cosine Similarity and Linear Discriminant Analysis* Cosine similarity is a simple measure of similarity between two vectors which consists in calculating the cosine of the angle between the two vectors.

In order to reduce intra-class variability and raise inter-class variability, discriminant analyses can be added to the back-end process. We supplemented the previous cosine similarity with a two-dimensional LDA, consisting in finding the orthogonal basis onto which the projection of x-vectors (extracted from our training groups) minimized intra-class variability while maximizing inter-class variability. The cosine similarity was then computed within this subspace.

*2.2.2.4.2. Probabilistic Linear Discriminant Analysis* Discriminant analysis can also be performed in a probabilistic way. PLDA was introduced in 2007 for face recognition (Prince, 2007) with i-vectors. We adapted it to PD detection, with x-vectors instead of i-vectors, and classes defined by the PD status of the participants (presence or absence of PD) instead of the speaker identity. We decomposed each x-vector **x** into: (i) an average component μ, computed on all the training subjects; (ii) a class-specific part **F.h**, which describes the inter-class variability; (iii) a speaker and session related part **G.w**, which describes the intra-class variability; (iv) and a residual term ϵ, assumed to be Gaussian with zero mean and diagonal covariance Σ (see Equations 1, 2).

(1) $$\begin{array}{l} \text{x}=\mu +\text{F.h}+\text{G.w}+\epsilon \end{array}$$

(2) $$\begin{array}{l} \text{with }\epsilon =N\left(0,\Sigma \right) \end{array}$$

The columns of matrix **F** provide a basis for the class-specific characteristics, with vector **h** the position of the subject in this subspace. The columns of matrix **G** provide a basis for the intra-class characteristics, with vector **w** the position of the speaker in this subspace. During the training phase, μ, **F**, **G**, and Σ are estimated. During the test phase, x-vectors of test subjects are compared to x-vector_*PD*_ and x-vector_*HC*_ by assessing the probability that they share the same class identity variable **h** (see Garcia-Romero and Espy-Wilson, 2011 for the details of the calculation method).

PLDA was preceded by an LDA in order to reduce the x-vector dimension.

##### 2.2.2.5. Validation and Ensemble Method

For the final classification and the validation we kept the ensemble method used for the MFCC-GMM analysis and described in section 2.2.1.4.

## 3. Results

In the following section we present the results of the x-vector analysis compared to the MFCC-GMM one for both genders and for both recording types (high-quality and telephone). We analyzed the effect of the audio segment durations, data augmentation, gender, type of classifier (for each speech task), dataset used for DNN training, and the choice of an ensemble method. More details about the MFCC-GMM analysis (men only) can be found in Jeancolas et al. (2019), in particular regarding the comparison of high-quality microphone vs. telephone recordings, as well as speech task effects. Performances were measured with the Equal Error Rate (EER), i.e., the error rate corresponding to the threshold for which false positive ratio is equal to false negative ratio (i.e., sensitivity equal to specificity), and Detection Error Tradeoff (DET) curves, using the Matlab software. The comparison between performances was expressed in percentage points (absolute difference).

### 3.1. Impact of Segment Duration

In order to have enough x-vectors for the LDA and PLDA training, we segmented our training audio files into 1–5 s segments. For the test phase, we compared two conditions. In the first condition, we considered a large variety of segment durations, from 25 ms to 100 s (in order to stay in the DNN compatible limits as explained in section 2.2.2.2). The durations of these test segments were not matched with the ones used for the DNN training (segment durations comprised between 2 and 4 s) nor with the ones used during our classifier training phase (durations from 1 to 5 s). In the second condition, we divided all our audio files into 1–5 s segments. Test segment durations were then matched with training segment durations. Results for both duration conditions, obtained from the sentence repetition tasks of male telephone recordings, are presented in Table 5 for the three classification methods (cosine similarity alone, with LDA, and PLDA). EER ranged from 36 to 41% for the condition with mismatched segments, and were improved by around 3% points for the condition with 1–5 s matched test segments (EER ranging from 32 to 39%), for the three classifiers. For the next experiments, we kept matched segment durations.

**Table 5:** PD vs. HC classification EER (in %) obtained with different segment lengths for the x-vectors extraction.

**Classifier**	**Mismatched**	**Matched**
x-vec + cos	41	**39**
x-vec + LDA + cos	36	**32**
x-vec + PLDA	36	**33**

*The classification was performed using the sentence repetition tasks of the male telephone recordings. [1–5 s] segments were used for training and either [25 ms–100 s] (mismatched) or [1–5 s] (matched) segments for test. X-vectors were classified in various ways: with cosine similarity (alone and with LDA) and with PLDA. Bold numbers indicate the best EER for each classifier*.

### 3.2. Comparison of Back-End Analyses

Classification of x-vectors with cosine similarity combined with LDA performed as well as PLDA, and were globally better than cosine similarity alone, whatever the recording condition (telephone or high-quality microphone) or speech task (see Tables 6, 7). These discriminant analyses led to a classification EER of up to 22% in males and 32% in females, with improvements of up to 15% in females, compared to cosine similarity alone. This improvement due to the discriminant analysis was observed in both genders but was predominantly sharper in women.

**Table 6:** PD vs. HC classification EER (in %) obtained with different classifiers: MFCC-GMM baseline, and x-vectors combined either with cosine similarity (alone and with LDA) or with PLDA, with and without data augmentation.

	**High-quality microphone**				**Telephone**			
	**Males**		**Females**		**Males**		**Females**	
	**Repet**	**Monol**	**Repet**	**Monol**	**Repet**	**Monol**	**Repet**	**Monol**
MFCC-GMM	**22**	26	42	45	35	36	42	40
x-vec + cos	32	35	51	41	39	33	49	43
x-vec + LDA + cos	**22**	27	39	32	**32**	35	**34**	34
x-vec + augLDA + cos	24	**25**	**34**	**30**	33	**33**	39	**33**
x-vec + PLDA	24	28	39	35	33	36	**34**	36
x-vec + augPLDA	25	**25**	**33**	**30**	**31**	**33**	37	**33**

*The datasets used are male and female high-quality microphone and telephone recordings. Analyzed tasks are free speech (monolog) and sentence repetitions (combined with readings for high-quality microphone recordings). Bold numbers indicate the best EERs for each dataset*.

**Table 7:** PD vs. HC classification EER (in %) obtained with different databases for the DNN training: the SRE16 database and our male telephone database (DDK tasks).

**Classifier**	**SRE16 DNN**	**Our DNN**
MFCC-GMM	**25**	**25**
x-vec + cos	**35**	47
x-vec + LDA + cos	**29**	**29**
x-vec + augLDA + cos	**30**	39
x-vec + PLDA	**30**	**30**
x-vec + augPLDA	**30**	38

*Bold numbers indicate the best EER for each classifier*.

### 3.3. Impact of Data Augmentation

In this section we assessed the impact of augmenting the LDA and PLDA training data. Results obtained with and without data augmentation for the LDA and PLDA training are detailed in Table 6 for the free speech and sentence repetition tasks and in Table 7 for the DDK task. We observed an improvement when using data augmentation for the free speech task for both genders, both types of microphone and both LDA and PLDA. In men, we obtained a 2–3% improvement with data augmentation for the free speech, compared to without data augmentation, leading to a respective EER of 25% with the high-quality microphone and 33% with the telephone. In women, the improvement ranged from 1% (with LDA from telephone recordings) to 5% (with PLDA from high-quality recordings) leading to respective EER of 33 and 30%. No consistent improvement due to data augmentation was found for sentence repetition tasks or DDK tasks.

### 3.4. X-Vectors vs. MFCC-GMM Comparison and Speech Task Influence

In this section we compared the classification methodologies using x-vectors with the more classic MFCC-GMM classification.

We already showed that data augmentation for the LDA and PLDA training improved classification for the free speech task but not for the text-dependent tasks. Therefore, for the comparison between MFCC-GMM and x-vectors, we used for the latter, cosine similarity combined with augmented LDA for the free speech task, and not augmented LDA for the sentence repetition and DDK tasks.

For both recording conditions and both genders, we observed improved classification performances with x-vectors, compared to MFCC-GMM, for the free speech task (see Table 6).

**Figure 7:**
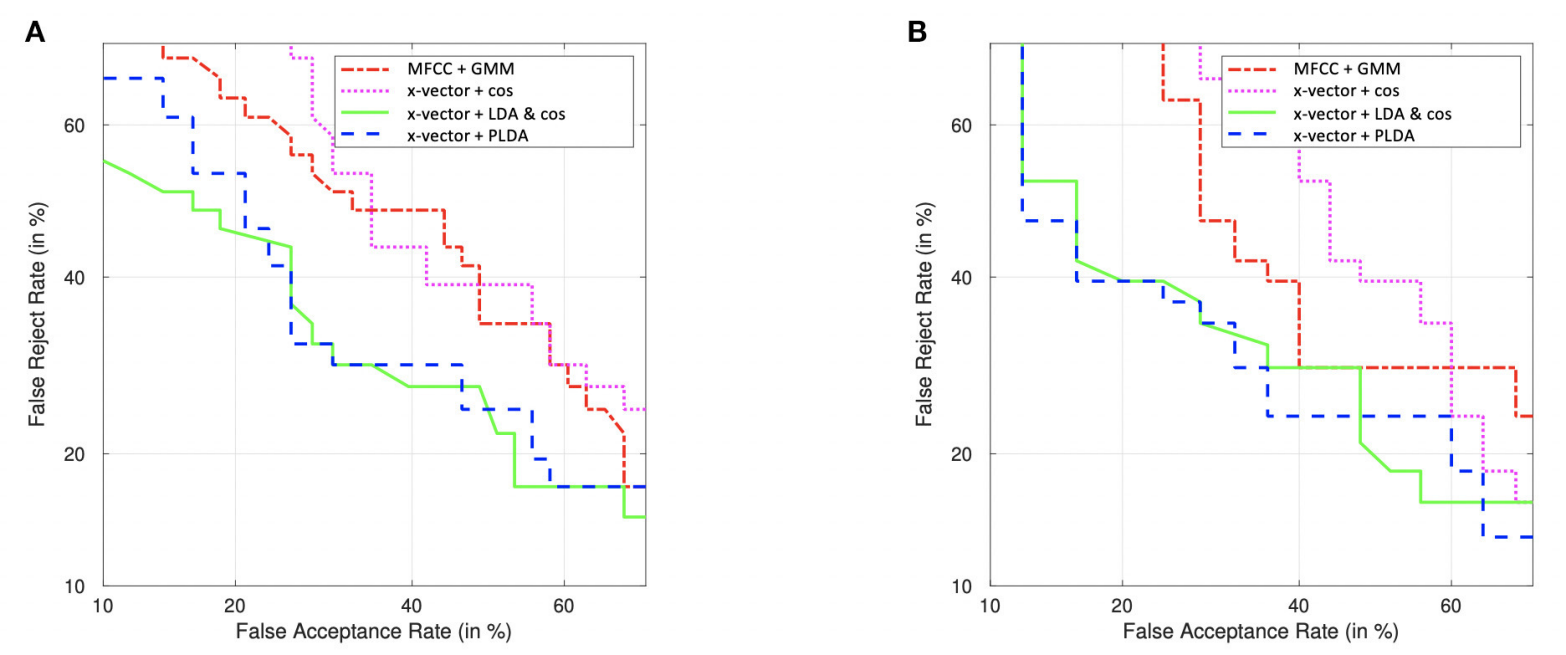
DET curves of female classification PD vs. HC, using the free speech task, recorded with the high-quality microphone **(A)** and with their telephone **(B)**. Comparison of classifiers performances: MFCC-GMM (baseline) and x-vectors combined either with cosine similarity (alone and with LDA) or with PLDA. LDA and PLDA are performed with data augmentation.

This improvement with x-vectors (compared to MFCC-GMM) was more pronounced in women (7% increase with telephone and 15% with high-quality microphone, compared to 1–3% in men). Detection Error Tradeoff (DET) curves in Figure 7 illustrate this classifier comparison in women.

Table 6 also shows an overall improvement with x-vectors for the sentence repetition and reading tasks but in a less consistent way.

Finally, results from the very specific DDK task (tested with male telephone recordings) are presented in Table 7. With this task, PD detection reached better performances using MFCC-GMM (EER = 25%) than with x-vectors (EER = 29–30%).

### 3.5. Gender Differences

MFCC-GMM and x-vector classifiers were trained separately for each gender, in order to study gender effect on early PD detection.

With the MFCC-GMM classification method, the female group showed poor PD detection performances: EER ranged from 40 to 45%, compared with 22–36% for men (see Table 6).

Interestingly, x-vectors when combined with a discriminant analysis (LDA or PLDA) clearly improved female classification performances, with an EER comprised between 30 and 39%. Nevertheless the female performances did not reach PD detection performances in males, whether obtained with the MFCC-GMM technique or with x-vectors (the best EER reached 22% with both methods in males).

### 3.6. Comparison With DNN Trained With Our Database

In order to make the DNN more suitable for the particular type of DDK tasks, we carried out an additional experiment, training this time the DNN with DDK tasks from our own database. The subjects used for the DNN training were the same as those used for the constitution of the average x-vector_*PD*_ and x-vector_*HC*_ and the LDA and PLDA training. The remaining subjects were used for the test. The results obtained are presented in Table 7. We noticed an 8–10% performance degradation when data augmentation was applied on the LDA and PLDA training. As for the results obtained with cosine similarity & LDA and with PLDA, without data augmentation, they were similar to those obtained with the previous pretrained DNN (EER ranging from 29 to 30%).

### 3.7. Aggregated Model vs. Single Model

In order to test the advantage of the ensemble method we used, we compared its performances with the results obtained with the corresponding single model. To estimate the performance of the single model, we fulfilled a classic random subsampling cross-validation. We averaged the DET curves from each run and calculated the EER corresponding to the average DET curve. We used male telephone recordings and considered the most appropriate tasks for each classifier. The performances obtained are detailed in Table 8 and compared to an excerpt from Table 6. With both MFCC-GMM and x-vector classifiers we observed a 2–3% improvement for the aggregated model, compared to the single model (EER = 32–33% for the aggregated model and 35% for the single model).

**Table 8:** PD vs. HC classification EER (in %) obtained with the aggregated model compared to the single model.

**Classifier**	**Task**	**Aggregated**	**Single**
MFCC-GMM	DDK	**25**	28
x-vec + LDA + cos	Repet	**32**	35
x-vec + augLDA + cos	Monol	**33**	35
x-vec + PLDA	Repet	**33**	35
x-vec + augPLDA	Monol	**33**	35

*Bold numbers indicate the best EER for each classifier*.

## 4. Discussion

According to the literature, the latest speaker recognition system, called x-vectors, provides more robust speaker representations and better recognition, when a large amount of training data is used. Our goal was to assess if this technique could be adapted to early PD detection (from recordings done with a high-quality microphone and via telephone network) and improve the detection performances. We compared a x-vector classification method to a more classic system based on MFCCs and GMMs.

We recorded 221 French speakers (PD subjects recently diagnosed and healthy controls) with a high-quality microphone and with their telephone. Our voice analyses were based on MFCC features. The baseline consisted in modeling the PD and HC distributions with two GMMs. For the x-vector technique, MFCCs were used as inputs of a feed-forward DNN from which embeddings (called x-vectors) were extracted then classified. Since DNN training usually requires a lot of data, we used a DNN trained on large speaker recognition databases. All the analyses were done separately for men and for women, in order to avoid additional variability due to gender, as well as to study a possible gender effect on early PD detection. We varied several experimental and methodological aspects in order to analyze their effect on the classification performances.

### 4.1. Influence of Segment Duration

We observed that using short audio segments that were matched between training and test provided better results (3% improvement). The improvement may be due to the matching durations between training segments and test segments, or to the fact that the classification was performed on more test segments (because they were shorter on average). This would compensate for the fact that taken separately, long segments have been shown to be better classified than short segments in speaker and language recognition (Snyder et al., 2017, 2018a).

### 4.2. Comparison of Back-End Analyses

We compared different back-end analyses used with x-vectors. We noticed that the addition of LDA clearly improved the cosine similarity classification and performed as well as a PLDA classifier. This can be explained by the fact that discriminant analyses reduce intra-class variance and increase inter-class variance, highlighting differences due to PD. This improvement due to the addition of discriminant analyses was even more pronounced in women (up to 15% improvement), whose voices are known to contain more variability (i.e., higher intra-class variance).

### 4.3. Influence of Data Augmentation

We found that augmenting data for the training of LDA and PLDA led to an improved classification for the free speech task (2–3% improvement) but not for text-dependent tasks (like sentence repetition and DDK). This can be explained by the fact that data augmentation, while increasing the training audio quantity, added phonetic variability which may have damaged the specificity of the phonetic content of the text-dependent tasks (like sentence repetitions, reading or DDK tasks). Data augmentation seems to be more suited for text-independent tasks (like free speech).

### 4.4. X-Vectors vs. MFCC-GMM Comparison and Speech Task Influence

The comparison with the MFCC-GMM classification showed that x-vectors performed better for the free-speech task, which is consistent with the fact that x-vectors were originally developed for text-independent speaker recognition. An overall improvement with x-vectors also appeared for the sentence repetition and reading tasks but in a less consistent way. This may be explained by the fact that GMMs captured well the specificity of text-dependent phonetic content. Indeed the reduction of phonetic content inter-subject variability made easier the isolation of the variability due to the disease, at least for the high-quality recordings. For telephone recordings there were no reading task, and the free speech task lasted much longer than sentence repetitions. This may compensate the expected improvement due to the constant phonetic content. Moreover, the participants carried out the telephone recordings by themselves without any experimenter to make them do the task again when not well executed. So mistakes or comments occurred during the telephone sentence repetitions, increasing a bit the variability of their phonetic content. As for x-vector classification, another aspect has to be taken into account. DNNs were trained with public databases with a very wide variability in the phonetic content, making the x-vector extractor not particularly suited to tasks with fixed phonetic content. Very specific tasks, like DDK, resulted in better performances with GMMs. Lower results with x-vectors for this task may be due to the DNN training, which was from recordings of conversations, containing wider variety of phonemes than DDK tasks (composed of vowels and stop consonants only). Thus, DDK specificity was not exploited by the DNN, resulting in a loss of discriminating power when using x-vectors.

### 4.5. Gender Effect

For all classifiers we noticed an important gender effect, with better performances for male PD detection. Several reasons may explain these gender differences. First of all, previous studies have reported wider female MFCC distributions, with more variability, making MFCC based classifications more difficult in women (Fraile et al., 2009). Tsanas et al. also noticed that MFCC features were more suited to monitor PD evolution in men than women (Tsanas et al., 2011). This may explain the worse classification performances with the MFCC-GMM classifier in women. X-vectors, combined with LDA or PLDA, handled the MFCC variability and led to a 7–15% improvement (compared to MFCC-GMM) for the classification in women. This was probably due to the fact that these discriminant analyses reduced intra-class variance, and thus tackled the MFCC variability issue in women. Nevertheless, even though LDA and PLDA reduced the classification performance gap between genders, it did not suppress it entirely. The remaining differences may be explained by other factors. First, a less pronounced brain atrophy (Tremblay et al., 2020) and less network disruptions (Haaxma et al., 2007) have been observed in the first stages of PD in women. In addition, the onset of symptoms is delayed on average by 2 years in women compared to men (Haaxma et al., 2007). A possible protective role of estrogen on PD has often been suggested to explain gender differences in early PD manifestations. Besides we can notice in our age-matched database a lower UPDRS III motor score in PD women as compared to PD men (see Tables 2, 3). A second factor possibly leading to gender differences in PD detection through voice, is that speech neural circuits have been reported to be different in men and women (Shaywitz et al., 1995; Dorion et al., 2000; Clements et al., 2006; de Lima Xavier et al., 2019; Jung et al., 2019). These circuits may therefore be differently affected in PD, leading to different types or degrees of vocal impairments.

### 4.6. Influence of the Dataset Used for the DNN Training

In order to make the DNN more specific to DDK tasks, we carried out an additional analysis by training it this time with our database (from DDK tasks). We noticed a clear performance degradation when data augmentation was applied on the LDA and PLDA trainings. This is consistent with the fact that data augmentation, while adding noise, impairs the specificity of the DDK phonetic content. Results obtained with cosine similarity + LDA and PLDA, without data augmentation, were similar to those obtained with the previous pretrained DNN. Our DNN training was certainly more specific but perhaps suffered from insufficient data quantity, which could explain why it did not outperform the pretrained DNN, confirming the importance of including a large quantity of data for the DNN training.

### 4.7. Influence of Ensemble Method

Finally, we observed a 2–3% improvement in the classification, when the ensemble method was used, for both MFCC-GMM and x-vectors classifiers. This demonstrates the interest of using ensemble methods for PD detection using voice.

### 4.8. Limitations

One of the limitations of this study is that our classifications were based only on cepstral features, which cannot capture all voice impairments due to PD. Indeed, articulatory impairments due to PD, like vowel dedifferentiation (due to an amplitude reduction of tongue and lips movements) and imprecise consonant articulation (e.g., vocal tract not completely closed during stop consonant pronunciations and bad coordination between laryngeal and supralaryngeal muscles) have an impact on the different spectral envelopes over time, so they are well captured by the different MFCC vectors. Nevertheless, MFCCs do not describe well several phonatory disruptions due to PD (such as pitch and intensity instability and voice hoarseness), nor abnormal pauses, or prosodic and rhythmic disruptions encountered in PD. For that, one should prefer global features to quantify them, as the ones stated in the introduction. A fusion of a classification based on these features, with the x-vector approach we presented in this paper, should improve the PD detection performances.

It is also worth highlighting that a comparison of our classifier performances with the literature remains difficult. Indeed, as far as we know, our results were the first obtained in early PD detection: (i) in women based only on voice; (ii) using recordings from the telephone channel (if we do not count our last conference paper on MFCC-GMM classification; Jeancolas et al., 2019); (iii) in French (if we still do not count; Jeancolas et al., 2019) and language has an impact on PD detection. A reliable comparison would require working on the same database, or at least with approximately the same number of subjects (with same gender), the same recording protocol, the same disease stage and the same language. Another aspect to take into account is the participant selection, and exclusion criteria. In our case, PD patients and controls were examined and selected by a neurologist, with definite inclusion and exclusion criteria, but in some studies they were self-selected without any checking of their medical condition (Arora et al., 2019). This has the advantage of facilitating the collection of big databases but has the inconvenient of less accurate labeling. Finally, our PD patients were recorded while they were on medication (ON-state), which reduced some speech impairments, making the classification more difficult than if they were on OFF-state.

An additional limitation of our work is that x-vectors were conceived for text-independent speaker recognition, whereas some of our tasks are text-dependent. Moreover, the use of complex artificial neural networks in the feature extraction process makes the reasons for score improvements difficult to understand and the physiopathology underlying PD speech impairments difficult to interpret. This fact affects the production and testing of new hypotheses.

Finally it would also be interesting to test other distance measures (such as the Euclidean or Mahalanobis distance or the Jensen-shannon divergence) to compare the x-vectors of the test subjects with the average x-vector_*PD*_ and x-vector_*HD*_. Indeed the cosine similarity we used is a very common metrics in speaker recognition, to compare x-vectors between themselves or even i-vectors, but might not be the most accurate metric in this case.

## 5. Conclusion and Future Works

The aim of the study was the discrimination between subjects with early stage Parkinson and healthy controls, thanks to a new speech analysis technique, adapted from recent findings in speaker recognition. We compared the efficacy of this method (called x-vectors) with the more classical MFCC-GMM technique, and varied several experimental and methodological aspects to determine the optimal approach.

We found that the x-vectors optimal methodological procedure for early PD detection consisted in using short and matched audio segments, adding discriminant analysis (LDA or PLDA) to the back-end process, augmenting the training data for the text-independent tasks, and using an ensemble method for the final classification. This resulted in better performances for early PD detection with x-vectors, compared with the MFCC-GMM technique, for the text-independent speech tasks. This improvement was observed for both genders, but this x-vector technique seems to be particularly suited to early PD detection in women, with 7–15% point improvement. The improved classification results with x-vectors, from text-independent tasks, were obtained with both professional microphone recordings and telephone recordings. This validated the x-vector approach for PD detection, using both high-quality recordings performed in a laboratory setting and low-quality recordings performed at home and transmitted through the telephone network.

In future work we will focus on other embeddings (e.g., d-vectors; Variani et al., 2014), which are also extracted using DNN trained with cepstral coefficients, but more suited to text-dependent tasks. We will also study high-level features related to other PD vocal disruptions, such as phonation, prosody, pause duration and rhythmic abilities, and combine them with this analysis (more related to articulation disorder), in order to gather all the information we can on early PD voice and improve the detection.

## Data Availability Statement

The datasets presented in this article are not readily available because of compliance with the ethical consents provided by the participants. Requests to access the datasets should be directed to Marie Vidailhet, marie.vidailhet@aphp.fr.

## Ethics Statement

The studies involving human participants were reviewed and approved by IRBParis VI, RCB: 2014-A00725-42. The patients/participants provided their written informed consent to participate in this study.

## Author Contributions

LJ: experimental design, data collection, data analysis and interpretation, and manuscript draft. DP-D: experimental design, validation of the analysis and its interpretation, and manuscript revision. GM: participants' diagnosis and clinical scores. B-EB: validation of the analysis and its interpretation. J-CC, MV, and SL: design and development of the ICEBERG study, data collection, and manuscript revision. HB: validation of the analysis and its interpretation and manuscript revision. All authors contributed to the article and approved the submitted version.

## Conflict of Interest

The authors declare that the research was conducted in the absence of any commercial or financial relationships that could be construed as a potential conflict of interest.

## Figures and Tables

**Figure A1:**
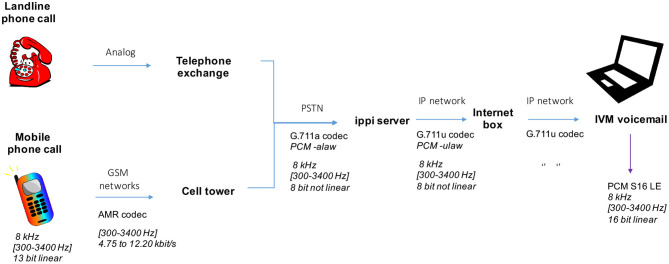
Transmission chain of the telephone recordings. GSM, Global System for Mobile Communications; AMR, Adaptive Multi-Rate; PSTN, Public switched telephone network; PCM, Pulse-code modulation; IP, Internet Protocol; IVM, interactive voicemail from NCH company.

## Annex

### Annex 1 : Vocal Task Content

#### High-Quality Recordings

Vocal tasks performed during the laboratory setting recordings and analyzed in the present study were: readings, sentence repetitions, a free speech and fast syllable repetitions. They were presented in a random order to the participants via a graphical user interface.

- **Reading**: a short text, a dialogue and 2 short sentences (1 question and 1 exclamatory sentence). The text contains all the phonemes of the French language. the dialogue and sentences were written in a common language level (with no subject-verb reversal) in order to emphasize prosody (which is impaired in PD).
  – *Text:*
    “Au loin un gosse trouve, dans la belle nuit complice, une merveilleuse et fraîche jeune campagne. Il n'a pas plus de dix ans et semble venir de trés loin. Comment il en est arrivé là, ça l'histoire ne le dit pas.”
  – *Dialogue:*
    — Tu as eu des nouvelles de Ludivine récemment? Elle ne répond plus à mes messages depuis quelques temps.
    — Je l'ai aperçue par hasard au parc hier. Tu ne devineras JAMAIS ce qu'elle faisait!
    — Vas-y raconte!
    — Elle courait autour du stade à CLOCHE-PIED et avec un BANDEAU sur les yeux!
    — Ha la la! Comment c'est possible de ne pas avoir peur du ridicule à ce point?
    — À mon avis elle aime juste bien se faire remarquer.
  – *Short sentences:*
  – Tu as appris la nouvelle?
  – C'est pas possible!
- **Sentence repetitions**: 2 questions and 2 exclamatory sentences given in an audio example. One of the two questions and one of the two exclamatory sentences were the same as for the reading (to allow for an exact comparison between reading and repetition).
  – “Tu as appris la nouvelle?”
  – “C'est pas possible!”
  – “Tu sais ce qu'il est devenu?”
  – “Il n'aurait jamais dû faire ça!”
- **Free speech**: participants were asked to talk about their day during one minute. This task provided a text-independent dataset.
- **Diadochokinesia (DDK)**: fast syllable repetitions without breathing (/pa/, /pu/, /ku/, /pupa/, /paku/, /pataka/, /badaga/, /patiku/, /pabiku/, /padiku/). All DDK tasks were performed once, except for /pataka/ which was performed twice. The consonants were stop consonants, because they are particularly impaired in PD. The vowels were those which form the vocalic triangle.

#### Telephone Recordings

Vocal tasks performed during the phone calls and analyzed in the present study were: sentence repetitions, a free speech and fast syllable repetitions. All the instructions were audio and given by the interactive voice server.

- **Sentence repetitions**: 3 questions and 3 exclamatory sentences with a common language level (with no subject-verb reversal) in order to emphasize the prosody (impaired in PD), as well as 2 declarative sentences for the comparison. Some sentences were the same as for the high-quality recordings.
  – “Tu as appris la nouvelle?”
  – “C'est pas possible!”
  – “Tu sais ce qu'il est devenu?”
  – “Il n'aurait jamais dû faire ça!”
  – “Tu as bien raison!”
  – “Comment il s'appelle déjà?”
  – “Les chiens aiment courir après les ballons.”
  – “Un carré est un rectangle particulier.”
- **Free speech**: participants were asked to talk about their day during one minute. This task provided a text-independent dataset.
- **Diadochokinesia (DDK)**: fast syllable repetitions without breathing (/pa/, /pu/, /ku/, /pupa/, /paku/, /pataka/). All DDK tasks were performed once. The consonants were stop consonants, because they are particularly impaired in PD.
